# Seasonal Variations in Hospital Admissions for ST-Elevation Myocardial Infarction in New Zealand

**DOI:** 10.4021/cr223e

**Published:** 2012-09-20

**Authors:** Janice Swampillai, Namal Wijesinghe, Cherian Sebastian, Gerard P Devlin

**Affiliations:** a Department of Cardiology, Waikato Hospital, New Zealand

**Keywords:** ST elevation myocardial infarction, Seasonal variation

## Abstract

**Background:**

Increased numbers of ST Elevation Myocardial Infarction (STEMI) admissions have been observed during winter in many countries. Our aim was to assess if seasonal variation of STEMI was present in the Waikato region of New Zealand.

**Methods:**

Case notes of patients admitted to Waikato hospital with STEMI between July 1998 and December 2007 were analysed. The incidence of STEMI during summer (December to February), autumn (March to May), winter (June to August) and spring (September to November) were calculated. The individuals were divided into 2 age groups of ≤ 70 and > 70 years of age.

**Results:**

A total of 3,569 patients (mean age 66.9 ± 14.1 years, 64% men) were included. STEMI presentation during winter was significantly higher compared with summer (35 ± 13 versus 27.3 ± 11.3 cases per month, P < 0.02) with 3 additional STEMI admissions per fortnight during winter months. The increase in STEMI in winter was more apparent in patients > 70 years of age, with an 8.5% increase in winter admissions compared to summer (P < 0.01). There was no significant difference in the incidence of STEMI between other seasons.

**Conclusion:**

There is a higher incidence of STEMI during winter in the Waikato region compared with summer. This increased incidence is particularly pronounced in patients over 70 years of age. Further investigations are necessary to elicit potential causes.

## Introduction

Winter months show an increased number of ST Elevation Myocardial Infarction (STEMI) admissions in many countries [1-3]. Stroke, pulmonary embolism and aortic dissection also show seasonal variations [4-6]. The exact reason is not clear but may relate to cold weather conditions, stress and increased inflammatory markers due to concurrent respiratory tract infections [7]. Some studies have also suggested that ambient temperature, air pollution, sunlight hours and air pressure may contribute to this phenomenon [8]. Our aim was to assess whether seasonal variation of STEMI exists in the Waikato region in New Zealand.

## Methods

We carried out a retrospective case note analysis of all patients admitted to Waikato hospital with STEMI between July 1998 and December 2007. Seasons were defined as summer (December to February), autumn (March to May), winter (June to August) and spring (September to November). The daily incidence of STEMI during summer, autumn, winter and spring was calculated. The number of days per month was standardised using the following equation: Standardised months = (number of cases/days in the month) × 30.

The individuals were divided into 2 age groups of ≤ 70 and > 70 years of age. Data on STEMI incidence distribution in the 4 seasons was analysed using the Chi squared test, with the probability value P < 0.05 considered to be statistically significant. Mean and minimum monthly temperatures were obtained from the National Institute of Water and Atmospheric Research.

## Results

**Figure 1:**
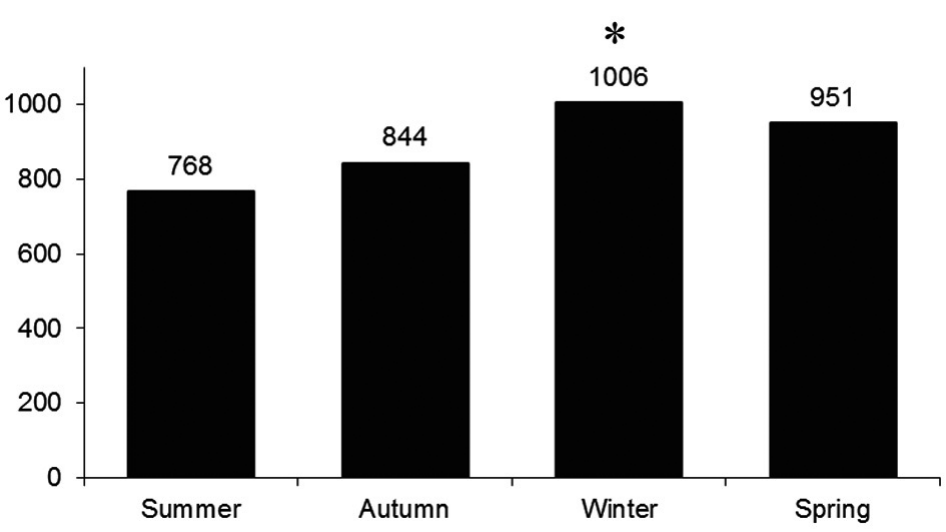
Total number of STEMI admissions during each season, *P < 0.01.

A total of 3,569 patients (mean age 66.9 ± 14.1 years, 64% men) were admitted to Waikato hospital with STEMI during the study period. The incidence of STEMI was highest in winter (Table 1), and STEMI presentation during winter was significantly higher compared with summer (35 ± 13 versus 27.3 ± 11.3 cases per month, P < 0.02) with 3 additional STEMI admissions per fortnight during winter months (Fig. 1).

**Table 1:** Seasonal Daily Incidence of STEMI in the 2 Age Groups

	Summer	Autumn	Winter	Spring
Total	0.95	1.02	1.14	1.05
Age ≤ 70	0.53	0.59	0.61	0.60
Age > 70	0.42	0.43	0.53	0.44

**Figure 2:**
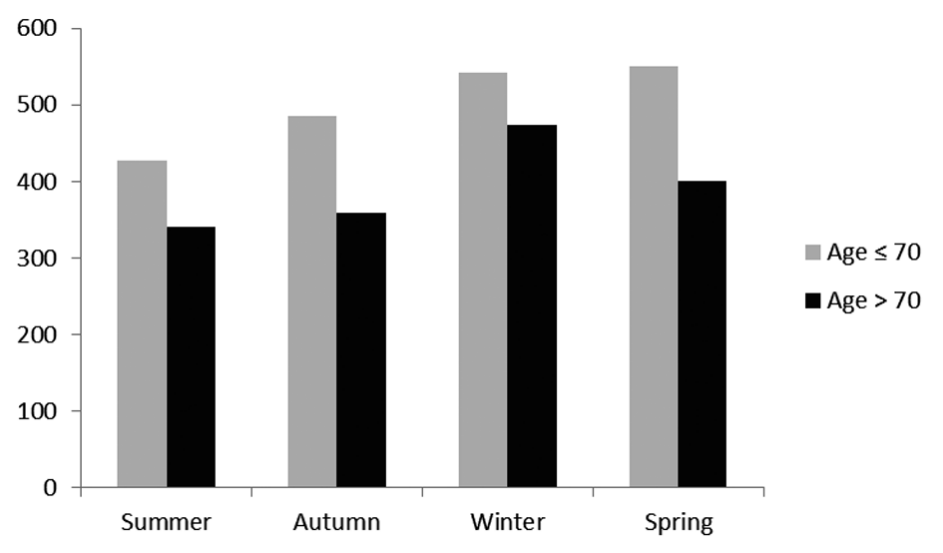
Total number of STEMI admissions in the 2 age groups.

The increase in STEMI in winter was more pronounced in patients > 70 years of age (Fig. 2), with an 8.5% increase in winter admissions compared to summer (P < 0.01). There was no significant difference in the incidence of STEMI between other seasons.

## Discussion

**Figure 3:**
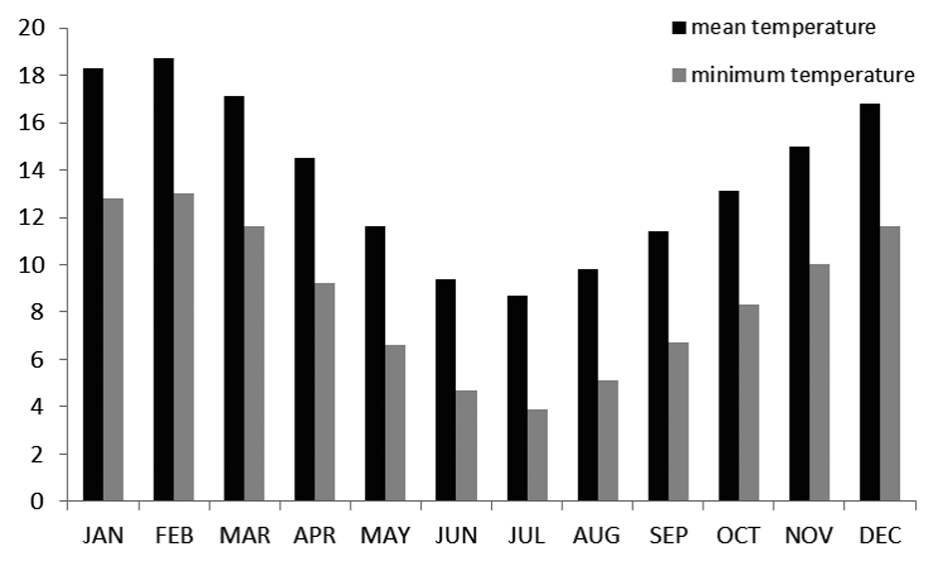
Monthly mean and minimum temperatures (°C) in the Waikato region.

The Waikato region in the North Island of New Zealand has a temperate climate (Fig. 3), and is situated at latitude 37.47 south, longitude 175.19 east, at 29 m of altitude. Minimum temperatures range from 13 °C in summer to 3.9 °C in winter, and mean sunshine hours vary from 231hours per month in summer to 103 hours per month in winter [9].

Our study confirms the seasonal variation of hospital admissions for STEMI that has been documented in both hemispheres [10, 11]. The first correlation between seasons and coronary thrombosis was documented in 1926 [12]. Although other authors have previously demonstrated a variation in coronary heart disease mortality in New Zealand [13], our study is the first to assess hospital admission rates.

We have demonstrated a 6.9% increase in STEMI admissions in winter compared to summer. This is comparable with seasonal changes observed in other parts of the world.

Winter weather is associated with a number of trigger factors for myocardial infarction, including increased sympathetic tone and blood pressure, myocardial oxygen demand, factors promoting coagulation, blood viscosity, cortisol and cholesterol levels [14]. External and lifestyle factors also contribute, as physical activity, fat and vitamin C intake, body mass index and smoking patterns have all been shown to alter in winter [15]. In addition to lower ambient temperatures, a reduction in hours of sunshine and vitamin D levels, environmental pollution and atmospheric pressure changes have also been implicated in the increase in myocardial infarctions in winter. Blood glucose and insulin levels are lower in summer in non-diabetic patients [16]. Acute and chronic infections, particularly respiratory tract infections also show an increased incidence in winter, and these concurrent infections are believed to increase infarction rates [7].

Reports from other parts of the world have shown that younger age groups show a spring peak in addition to a winter peak, and we have also observed this trend from our data (Fig. 2) [17]. Winter peaks in mortality from coronary heart disease increase with age. In our population, there was an 8.5% increase in STEMI admissions in the over 70 year old age group in winter compared to summer. Environmental factors are especially important in older people, and ineffective heating and insulation, lack of warm clothing, poor nutrition and decreased physical activity are likely to be influential.

This study is important to establish that there are seasonal variations in STEMI admission in the Waikato region. The focus of attention should now be on identifying potentially avoidable triggers in the winter months. In particular, patients with coronary disease should be aware of this seasonal trend, and measures taken to ensure that their lifestyles are as healthy in winter as in summer. The United Kingdom Meteorological Office health forecasting services have been developed in order to limit the impact of weather on people's health and hospital admissions. Healthy Outlook is a preventative service aimed at helping people with chronic obstructive pulmonary disease manage their condition. When particular climatic conditions such as extremes of temperature and humidity are expected, the Met Office's Healthy Outlook service sends an automated phone message to patients, directing them to follow simple health advice designed to help them stay well. This resource enables patients to take appropriate action and plan ahead to reduce the impact of a cold snap that could result in an admission to hospital. A Met Office evaluation found an average national reduction in hospital admissions of 20% for those using the scheme [18]. A similar resource could potentially be beneficial in patients with or at risk for coronary heart disease, although this would not impact on those presenting for the first time with a myocardial infarction. Simple safeguards such as wearing warm clothing indoors and outdoors, installing suitable heating and maintaining a healthy diet could help to reduce the incidence of hospital admissions for STEMI in winter. The impact of climate change on seasonal variations of myocardial infarction may influence these patterns in the future.

### Limitations

We have only analysed data from patients admitted to hospital with primary diagnosis of STEMI in our region. As a result we have not included all events in the community. This data does not take into account patients who were hospitalised for other reasons and developed a STEMI during the course of the admission. In addition we have not distinguished between initial and recurrent STEMI, and these may show different seasonal variations.

### Conclusion

There is a higher incidence of STEMI during winter in the Waikato region compared with summer. This increased incidence is particularly pronounced in patients over 70 years of age. Further investigations are necessary to elicit potential causes.
